# Combination of Cardiovascular, Kidney, and Metabolic Diseases in a Syndrome Named Cardiovascular-Kidney-Metabolic, With New Risk Prediction Equations

**DOI:** 10.1016/j.ekir.2024.05.033

**Published:** 2024-06-06

**Authors:** Ziad A. Massy, Tilman B. Drueke

**Affiliations:** 1 Inserm Unit 1018, Team 5, CESP, Hôpital Paul Brousse, Paris-Sud University (UPS) and Versailles Saint-Quentin-en-Yvelines University (Paris-Ile-de-France-Ouest University, UVSQ), Villejuif, France; 2 Association pour l’Utilisation du Rein Artificiel dans la région parisienne (AURA), Paris, France; 3 Department of Nephrology, Ambroise Paré University Hospital, APHP, Boulogne-Billancourt, Paris, France

**Keywords:** cardiovascular disease, CKM syndrome, kidney disease, metabolic syndrome, risk prediction

## Abstract

Associations of chronic kidney disease (CKD) with metabolic syndrome and cardiovascular disease (CVD) have long been recognized. Until recently, such associations were mainly limited to interrelationships between either heart and kidney, heart and metabolic syndrome, or metabolic syndrome and kidney. It is the merit of the American Heart Association (AHA) to have set up a work group of cardiologists, endocrinologists, and nephrologists for the purpose of combining all 3 disorders in a single entity, as an appreciation of their pathophysiological interrelatedness. To this end, they proposed the term cardiovascular-kidney-metabolic (CKM) syndrome, which reflects multidirectional relationships among metabolic risk factors, CKD, and the cardiovascular system. Following a consensus approach in defining CKM with 5 stages, the work group subsequently developed new risk prediction equations, named predicting risk of CVD events (PREVENT) equations, which included estimated glomerular filtration rate (eGFR) and albuminuria as variables in addition to traditional cardiovascular and metabolic factors. Despite several limitations, this development is a major step forward in cardiovascular risk prediction. Its clinical application should translate into earlier, more appropriate treatment and prevention of CKM syndrome.

It has long been known that CKD is frequently observed in association with metabolic syndrome, CVD, or both. Determining the directionality of the relationship is sometimes easy. As an example, in patients with primary forms of chronic glomerulonephritis, CVD is most often secondary to kidney disease. On the contrary, in patients with metabolic syndrome, diabetes, or CVD, the development of kidney disease is most often a secondary event. However, in many instances the sequence of events is more difficult, if not impossible to establish. In any case, one would always like to know which came first, the egg or the chicken. The answer to this question is of importance not only from a theoretical, pathophysiological point of view, but also from a practical, clinical point of view. Better knowledge can lead to improved individualized disease management.

### Cardiorenal Syndrome

The interrelationship between heart and kidney disease has been named "cardiorenal syndrome." The creation of this term dates back to 2009 when it was first officially defined at a consensus conference of the Acute Dialysis Quality Initiative.^1^ The authors' aim was to characterize and classify the various interrelationships between acute and chronic heart and kidney diseases. The consensus conference defined 5 forms of heart-kidney interaction; including type 1, in which acute heart failure is directly associated with acute kidney injury; type 2, in which chronic heart failure is associated with CKD; type 3, in which acute kidney injury is associated with acute heart failure; type 4, in which the driving factor of CKD is associated with chronic heart failure; and type 5, in which there is concomitant development of both kidney and heart failure. Cardiorenal syndrome has been more recently defined in a simplified way by the North American National Heart, Lung, and Blood Institute, as a result of interactions between the kidneys and other circulatory compartments that increase circulating volume, which exacerbates the symptoms of heart failure and disease progression, encompassing a variety of disorders involving both the heart and kidneys, whereby acute or chronic dysfunction of 1 organ may induce acute or chronic dysfunction in the other organ.^2^

Although the term cardiorenal syndrome has been widely adopted, it is interesting to note that its current classification and even the appropriateness of the term itself have recently been challenged by Zoccali *et al.*^3^ The authors posit that the term "disorder" is more appropriate than the term "syndrome" to describe concomitant cardiovascular and kidney dysfunction and/or damage. Whatever the optimal term to be used, the authors agreed with the appropriateness of this conceptual framework because it builds upon the fact that CVD and CKD share common risk factors and underlying pathophysiologic mechanisms.

### Metabolic Syndrome and Kidney Disease

An interrelationship between metabolic syndrome and kidney disease has been known for at least 20 years.^4^, ^5^, ^6^ In a cross-sectional study of the Third National Health and Nutrition Examination Survey in the US, Chen *et al.*^4^ included adult participants in CKD (*n* = 6217) and microalbuminuria (*n* = 6125) analyses. Compared with participants without metabolic syndrome, those with the syndrome had multivariate-adjusted odds ratios of CKD and microalbuminuria of 2.60 (95% confidence interval: 1.68–4.03) and 1.89 (95% confidence interval: 1.34–2.67), respectively. In 10,096 nondiabetic individuals with normal baseline kidney function who participated at the Atherosclerosis Risk in Communities study in the US, Kurella *et al.*^5^ reported a multivariable-adjusted odds ratio of developing CKD of 1.43 in participants with metabolic syndrome, compared with participants having no traits of metabolic syndrome. Bonnet *et al.*^6^ studied 2738 subjects from the Epidemiological Study on the Insulin Resistance Syndrome cohort without microalbuminuria or diabetes at baseline. At 6 years of follow-up, they found that 254 individuals (9.3%) had developed albuminuria ≥ 20 mg/l, which was significantly and positively associated with waist circumference (reflecting abdominal adiposity) and blood pressure, although not with fasting glucose, lipids or body mass index (BMI) in either sex.^6^

Given the steadily increasing rate of overweight, obesity, and type 2 diabetes worldwide, although with marked discrepancies between geographical regions, the coexistence of these conditions with heart failure and CKD is striking.^7^ It clearly needs special attention and multidisciplinary approaches.

### CKM syndrome

In this vein, as an extension beyond the cardiorenal and metabolic-renal connections, the American Heart Association (AHA) has recently proposed a broader concept in the form of a unifying syndrome. On behalf of the AHA, Ndumele *et al.*^8^ named this entity CKM syndrome, as an appreciation of the pathophysiological interrelatedness of the following: (i) metabolic risk factors such as obesity and diabetes, (ii) CKD, and (iii) CVD. In Figure 1, we show a conceptual diagram for CKM syndrome with its pathophysiological consequences reflecting multidirectional relationships among metabolic risk factors, CKD, and the cardiovascular system.

**Figure 1:**
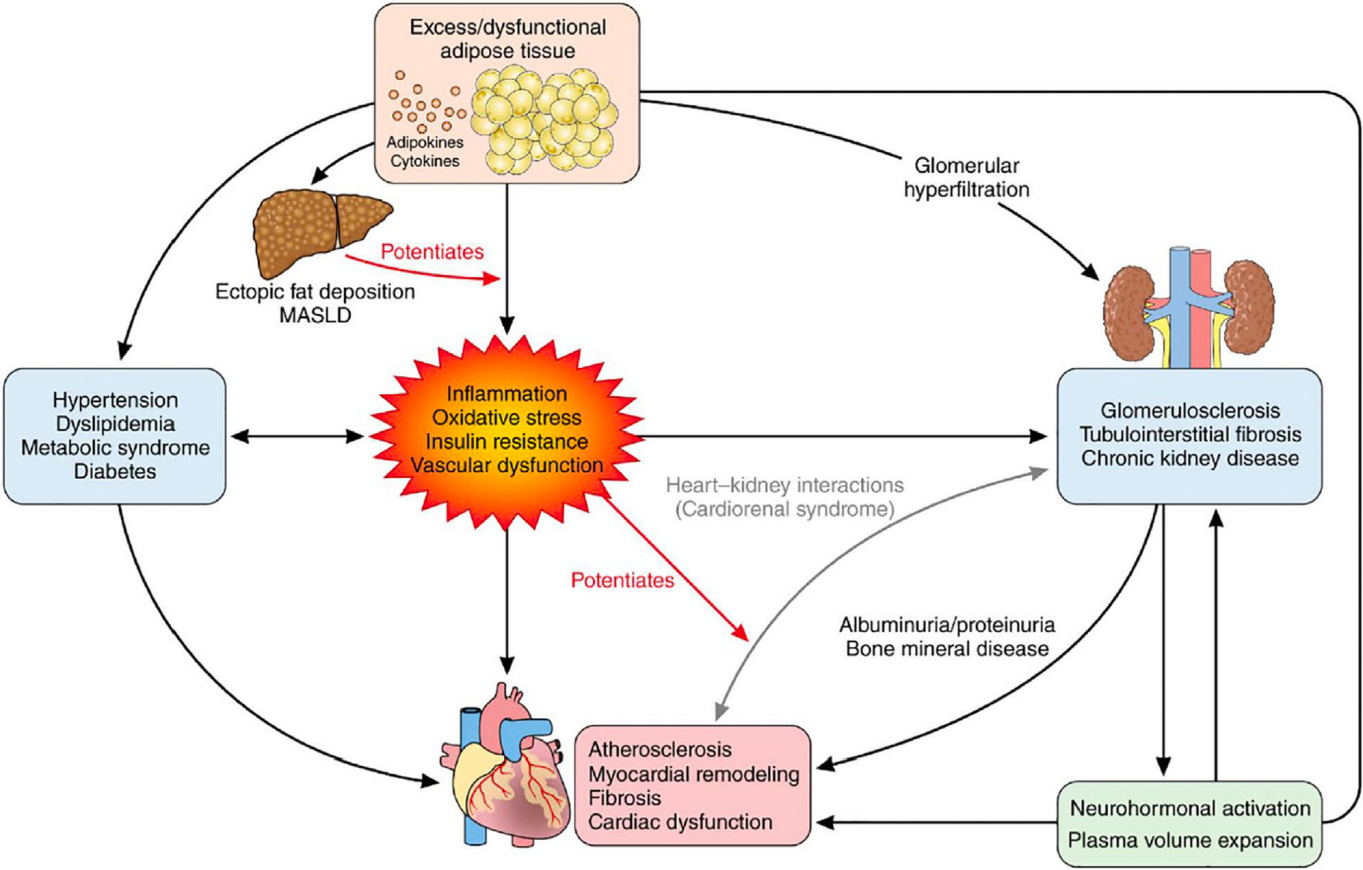
Conceptual diagram for CKM syndrome. The image displays the pathophysiology underlying CKM syndrome. CKM syndrome, most commonly, originates from excess adipose tissue, dysfunctional adipose tissue, or both. Multiple pathological processes related to dysfunctional adipose tissue result in insulin resistance and eventual hyperglycemia. Inflammation, oxidative stress, insulin resistance, and vascular dysfunction are highlighted as central processes leading to the development of metabolic risk factors, progression of kidney disease, potentiation of heart-kidney interactions, and development of cardiovascular diseases. Metabolic risk factors and chronic kidney disease further predispose to cardiovascular diseases through multiple direct and indirect pathways. CKM, cardiovascular-kidney-metabolic; MASLD, metabolic dysfunction-associated steatotic liver disease.

The reason for creating the new term is that CKM syndrome proposes an integrated staging system. The staging encompasses individuals at risk for CVD due to the presence of metabolic risk factors, hypertension, diabetes, or CKD, or a combination of these; as well as individuals with existing CVD that is potentially related to or complicates metabolic risk factors or CKD.^9^ Individuals who do not present CKM health risk factors are classified as stage 0. Those with excess and/or dysfunctional adiposity are classified as stage 1, those with metabolic risk factors and CKD as stage 2, those with subclinical CVD in CKM as stage 3, and those with clinical CVD in CKM as stage 4. The complete definitions of CKM health stages 0 through 4 are outlined in Table 1.

**Table 1:** Definitions of CKM health stages

CKM health stages	Definition
Stage 0: No CKM health risk factors	Individuals without overweight/obesity, metabolic risk factors (hypertension, hypertriglyceridemia, MetS^a^, diabetes), CKD, or subclinical/clinical CVD
Stage 1: Excess and/or dysfunctional adiposity	Individuals with overweight/obesity, abdominal obesity, or dysfunctional adipose tissue, without the presence of other metabolic risk factors or CKD BMI ≥ 25 kg/m^2^ (or ≥23 kg/m^2^ if Asian ancestry) Waist circumference ≥88/102 cm in women/men (or if Asian ancestry, ≥80/90 cm in women/men) and/or Fasting blood glucose ≥100–124 mg/dl or HbA1c between 5.7% and 6.4%^b^
Stage 2: Metabolic risk factors and CKD	Individuals with metabolic risk factors (hypertriglyceridemia (≥135 mg/dl), hypertension, MetS, diabetes) or CKD
Stage 3: Subclinical CVD in CKM	Subclinical ASCVD or subclinical HF among individuals with excess/dysfunctional adiposity, other metabolic risk factors, or CKD Subclinical ASCVD to be principally diagnosed by coronary artery calcification (subclinical atherosclerosis by coronary catheterization/CT angiography also meets criteria) Subclinical HF diagnosed by elevated cardiac biomarkers (NT-proBNP ≥ 125 pg/ml, high-sensitivity troponin T ≥ 14 ng/l for women and ≥22 ng/l for men, high-sensitivity troponin I ≥10 ng/l for women and ≥12 ng/l for men) or by echocardiographic parameters, with combination indicating highest HF risk Risk equivalents of subclinical CVD Very high-risk CKD (G4 or G5 CKD or very high risk per KDIGO classification) High predicted 10-y CVD risk
Stage 4: Clinical CVD in CKM	Clinical CVD (coronary heart disease, heart failure, stroke, peripheral artery disease, AFib) among individuals with excess/dysfunctional adiposity, other metabolic risk factors, or CKD Stage 4a: no kidney failure Stage 4b: kidney failure present

ASCVD, atherosclerotic cardiovascular disease; BMI, body mass index; CKD, chronic kidney disease; CKM, cardiovascular-kidney-metabolic; CT, computed tomography; CVD, cardiovascular disease; HbA1c, hemoglobin A1c; HDL, high-density lipoprotein; HF, heart failure; hs-troponin, high-sensitivity troponin; KDIGO, Kidney Disease Improving Global Outcomes; MetS, metabolic syndrome; and NT-proBNP; N-terminal pro-B-type natriuretic peptide.

a MetS is defined by the presence of 3 or more of the following: (i) waist circumference ≥ 88 cm for women and ≥102 cm for men (≥80 cm for women and ≥90 cm for men if of Asian ancestry); (ii) HDL cholesterol < 40 mg/dl for men and <50 mg/dl for women; (iii) triglycerides ≥ 150 mg/dl; (4) elevated blood pressure (systolic blood pressure ≥ 130 mm Hg or diastolic blood pressure ≥ 80 mm Hg and/or use of antihypertensive medications); and (5) fasting blood glucose ≥ 100 mg/dl.

b Individuals with gestational diabetes should receive intensified screening for impaired glucose tolerance after pregnancy.

The underlying mechanisms comprise a variety of interconnected factors.^9^ These factors include hyperglycemia, insulin resistance, stimulation of the renin-angiotensin-aldosterone system, enhanced generation of advanced glycation end-products, oxidative stress, dyslipidemia and lipotoxicity, mineral disturbances, endoplasmic reticulum stress, mitochondrial dysfunction with impaired cellular energy production, chronic (micro)inflammation, and potentially uremic toxins.

According to Ndumele *et al.*^8^ CKM syndrome most commonly originates from excess or dysfunctional adipose tissue or both, with an excessive release of proinflammatory and prooxidative products. This in turn leads to impaired insulin sensitivity and glucose intolerance, damage of the cardiovascular system and the kidney, and hepatic steatosis. Ectopic fat can also release locally active mediators and produce compressive organ damage in heart and kidney, inducing arrhythmias, myocardial and coronary artery disease, and arterial hypertension.^10^, ^11^, ^12^, ^13^ In Table 2, we show a list of risk-enhancing factors for CKM syndrome.^14^ They encompass an impressive diversity of predisposing conditions.

**Table 2:** Risk-enhancing factors for CKM syndrome^a^

Chronic inflammatory conditions (e.g., psoriasis, RA, lupus, HIV/AIDS)
High-risk demographic groups (e.g., South Asian ancestry, lower socioeconomic status)
High burden of adverse SDOH
Mental health disorders (e.g., depression and anxiety)
Sleep disorders (e.g., obstructive sleep apnea)
Sex-specific risk enhancers (beyond gestational diabetes consideration in stage 1) History of premature menopause (age < 40 y) History of adverse pregnancy outcomes (e.g., hypertensive disorders of pregnancy, preterm birth, small for gestational age) Polycystic ovarian syndrome Erectile dysfunction
Elevated high-sensitivity C-reactive protein (≥2.0 mg/l, if measured)
Family history of kidney failure; family history of diabetes

RA, rheumatoid arthritis; SDOH, social determinants of health.

a These factors increase the likelihood of progression along CKM stages with associated risk for cardiovascular disease and kidney failure.

The pathophysiological consequences of these processes are numerous, including disturbances of endothelial function, atherosclerosis, thrombosis, as well as cardiac and kidney dysfunction and fibrosis. All these processes favor the development of cardiovascular, cerebrovascular, peripheral artery, and kidney disease. Underlying mechanisms can be broadly classified as hemodynamic, metabolic, inflammatory, and fibrotic.

Nevertheless, critical knowledge gaps remain regarding the mechanisms of disease development, the influence of heterogeneous clinical phenotypes, the role of various health determinants and risk factors, as well as the assessment of disease incidence in the context of competing risks, as pointed out by Ndumele *et al.*^8^ The authors stress that CKM syndrome severity and related adverse outcomes are also influenced by unhealthy lifestyle and insufficient self-care, which are amenable to correction by appropriate policy changes, improvements in economic conditions, and environmental changes.

Although Cartesian logic pushes us to consider that using the term CKM disorder would be preferable to that of CKM syndrome, in agreement with the above-mentioned term cardiorenal disorder,^3^ the concept of this new entity should prove to be useful for both clinical management and research purposes.

From the nephrology perspective, the inclusion in CKM syndrome of the 2 major parameters of kidney function, namely glomerular filtration rate and albuminuria, is an important change made to a hitherto mainly cardiology-orientated view of patient outcomes driven by CVDs.

Taking into account CKD with its dramatic increase in cardiovascular events, in particular heart failure, arrhythmias, and sudden cardiac death as major risk components, is an important addition to the risk conferred by atherosclerotic CVD in the general population.^15^ It is noteworthy that in patients with kidney failure, a large number of nontraditional, nonischemic risk factors strongly contribute to the occurrence of cardiovascular and cerebrovascular events, including iso-osmotic and nonosmotic sodium retention, volume expansion, anemia, inflammation, malnutrition, sympathetic overactivity, mineral bone disorders, accumulation of a class of endogenous compounds called “uremic toxins,” and a variety of hormonal disorders.^15^, ^16^, ^17^ It has been known for decades that increasing CKD severity associates with a progressive increase in the incidence of cardiovascular events. Go *et al.*^18^ showed 20 years ago that patients with an eGFR of 45 to 59 ml/min per 1.73 m^2^ (CKD stage 3a) had a 3.6-fold increase in age-standardized rates of cardiovascular events as compared to an eGFR ≥ 60 ml/min per 1.73 m^2^ (CKD stages 1–2), and a 36.6-fold increase at eGFR < 15 ml/min per 1.73 m^2^ (CKD stage 5). In Figure 2, we illustrate the dramatic increase conferred by kidney failure, and concomitantly, a similar steep increase in hospitalization and mortality rates. In keeping with the study by Go *et al.*,^18^ a recent meta-analysis performed in more than 27 million individuals from 114 global cohorts confirmed and extended this finding, in that lower eGFR was associated with increased rates of 10 adverse outcomes, including risk of kidney failure requiring kidney replacement therapy, all-cause mortality, cardiovascular mortality, acute kidney injury, any hospitalization, coronary heart disease, stroke, heart failure, atrial fibrillation, and peripheral artery disease.^19^

**Figure 2:**
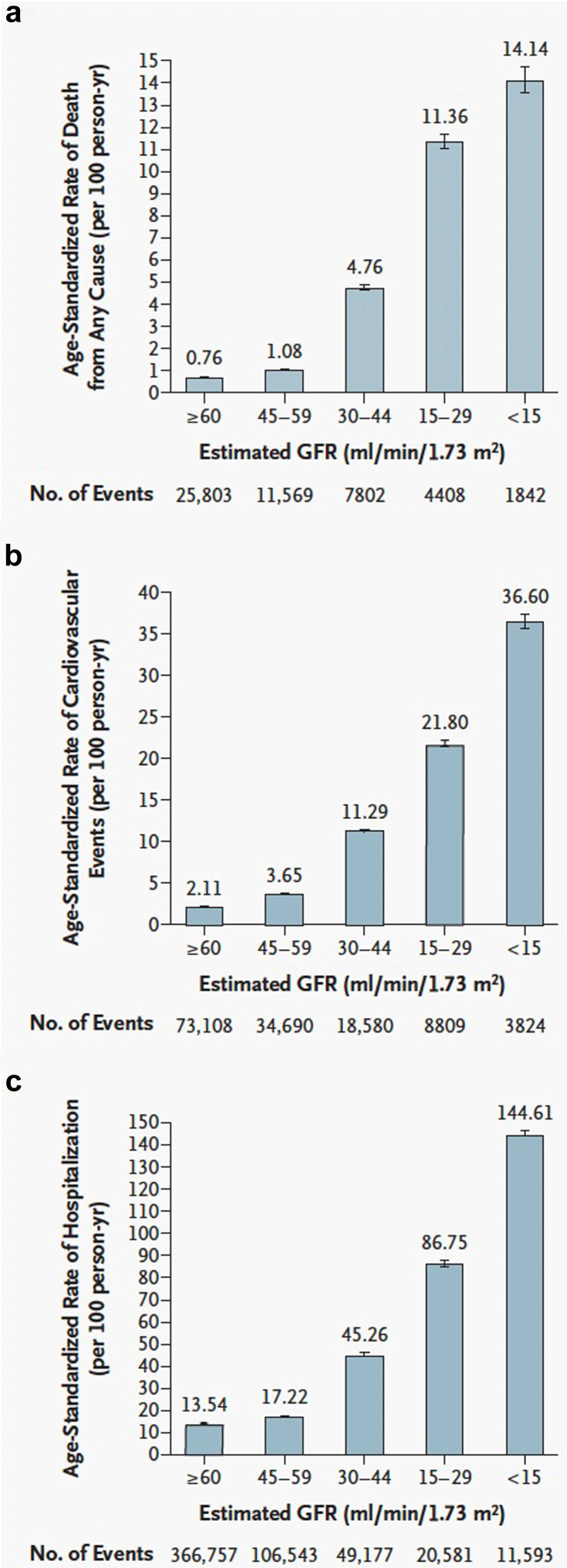
Age-standardized rates of death from (a) any cause, (b) cardiovascular events, and (c) hospitalization, according to the estimated GFR among 1,120,295 ambulatory adults. A cardiovascular event was defined as hospitalization for coronary heart disease, heart failure, ischemic stroke, and peripheral arterial disease. Error bars represent 95 percent confidence intervals. The rate of events is listed above each bar. GFR, glomerular filtration rate.

Considering albuminuria in the definition of CKM is important as well. In the same recent report, a second meta-analysis based on more than 9 million individuals from same 114 global cohorts showed that more severe urinary albumin-to-creatinine ratio (uACR) was associated with increased rates of all 10 adverse outcomes.^19^ The incremental increase of all these complications with worsened categories of both eGFR and albuminuria has found an elegant pictorial illustration in the recent Kidney Disease: Improving Global Outcomes Clinical Practice Guideline for the Evaluation and Management of CKD,^20^ as shown in Figure 3.

**Figure 3:**
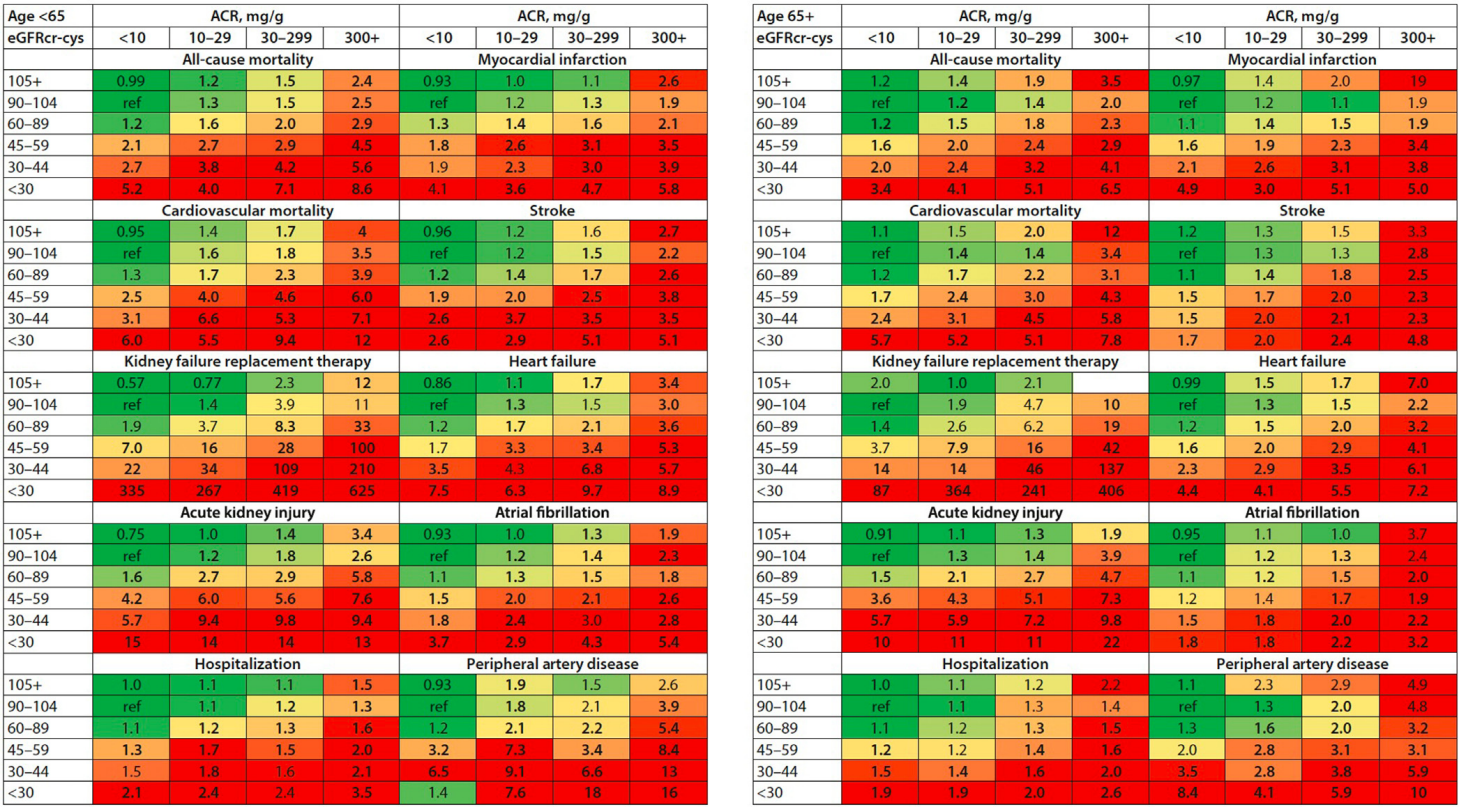
Associations of chronic kidney disease (CKD) staging by estimated glomerular filtration rate by creatinine and cystatin C (eGFRcr-cys) and albumin-to-creatinine ratio (ACR) categories and risks for 10 common complications by age in multivariable-adjusted analyses. Numbers reflect the adjusted hazard ratio compared with the reference cell. Adjustment variables included age; sex; smoking status (current, former, or never); systolic blood pressure; total cholesterol; high-density lipoprotein cholesterol; body mass index; use of antihypertensive medications; and a medical history of diabetes, coronary heart disease, stroke, heart failure, atrial fibrillation, peripheral artery disease, cancer, and chronic obstructive pulmonary disease, where relevant. The colors were determined for each outcome separately using the following rule: the percentile shaded the darkest green color corresponds to the proportion of cells in the grid without CKD (e.g., 6 of 24 cells), and the percentile shaded the darkest red color corresponds to proportion expected to be at highest risk (e.g., 5 of 24 cells). In this manner, the numbers of green and red cells are consistent across outcomes, but the patterns are allowed to differ. ref, reference cell.

### Risk Prediction and Risk-Based Prevention Guidelines

Existing risk predictions and risk-based prevention guidelines, more or less, focus specifically on risks linked to disease categories such as CVD, diabetes, hypertension, metabolic syndrome, or kidney disease. These predictions and guidelines have, most often, been issued by national or international medical specialty organizations. Here are some examples, without providing an exhaustive list.

In the US, the most recent AHA/American College of Cardiology Guideline Recommendations for Multivariable Risk Assessment and Risk-Based Prevention were issued in the years 2018 to 2022, based on pooled cohort equations. The recommendations were devoted to the management of high blood pressure,^21^ the management of cholesterol,^22^ the primary prevention of CVD,^23^ and the management of heart failure.^24^

The American Diabetes Association 2023 Standards of Care endorsed the use of pooled cohort equations for the assessment of atherosclerotic CVD risk among individuals with diabetes.^25^

The American Association of Clinical Endocrinology developed guidelines for the management of patients with diabetes mellitus in 2023, consisting of visual guidance based on concise graphic algorithms to assist with clinical decision-making of health care professionals.^26^

Guidelines for the medical care of patients with obesity were developed by the American Association of Clinical Endocrinologists Board of Directors and the American College of Endocrinology Board of Trustees in 2016.^27^

A number of heart failure risk scores were developed to predict 10-year risk of new-onset heart failure in the general population, notably 2 from the Atherosclerosis Risk in Communities study using 11 clinical characteristics or demographics with or without the cardiac biomarker N-terminal brain natriuretic peptide; both performed comparably.^28^

The 2021 European Society of Cardiology guideline on CVD prevention previously categorized moderate and severe CKD as high and very-high CVD risk status, based on traditional cardiovascular risk factors, that is, diabetes, hypertension, dyslipidemia, and smoking. Specifically, it developed systemic coronary risk estimation 2 and systemic coronary risk estimation 2 in older persons to predict CVD risk. It included neither age *per se*, nor eGFR or albuminuria in its algorithms. The authors subsequently developed and validated an “add-on” to incorporate CKD measures (eGFR, uACR, and dipstick proteinuria) into these algorithms, using a validated approach. The add-ons with CKD measures improved CVD risk prediction beyond systemic coronary risk estimation 2 and systemic coronary risk estimation 2 in older persons.^29^

As far as CKD is concerned, multiple national and international guidelines have been issued. The most recent international clinical practice guideline for the evaluation and management of CKD has been published by the Kidney Disease: Improving Global Outcomes CKD Work Group in March 2024.^20^ The work group developed an update from the previous guideline on CKD, demonstrated associations between CKD staging by eGFR and uACR categories with risks for 10 common complications in multivariable-adjusted analyses, and issued treatment approaches and guideline recommendations based on systematic reviews of relevant studies.

### New Risk Prediction Equations Based on CKM Syndrome

Following its consensus approach in defining CKM with its 5 stages, the AHA undertook to develop new risk prediction equations, including eGFR and albuminuria as variables in addition to traditional cardiovascular and metabolic variables. This further development was deemed necessary because of changes in the prevalence of risk factors such as less tobacco use and lower lipid serum levels in recent years, changes in care patterns such as a more widespread use of antihypertensive agents, and the risk of overestimating incident atherosclerotic CVD with pooled cohort equations.^30^ Although epidemiological data demonstrated higher absolute risk of both atherosclerotic CVD and heart failure with progression from CKM stage 0 to stage 3 more refined, optimal strategies for absolute risk assessment and primary prevention still had to be set up, all the more because a growing number of therapies have become available for all 3 CKM components in recent years.

Consequently, Khan *et al.*^31^ developed and validated new, sex-specific and race-free risk equations, named PREVENT equations, among US adults aged 30 to 79 years, who are without known CVD. The development was based on a derivation sample of individual-level participant data from 25 data sets with over 3 million participants and followed by an external validation performed in over 3 million participants from 21 additional data sets. Data sets were included if they were US-based and had measured data on the following 5 key risk factors of interest: systolic blood pressure, total cholesterol, high-density lipoprotein cholesterol, BMI, and eGFR. Based on the derivation sample, the PREVENT equations provide CVD risk estimates over time periods of 10 and 30 years, respectively. They include eGFR as a risk predictor and adjust for competing risk of non-CVD death. When clinically indicated, additional models include other CKM factors such as uACR, hemoglobin A1c, and social determinants of health. The median C-statistics in external validation for CVD were 0.794 and 0.757, and the calibration slopes 1.03 and 0.94 in males and females, respectively. A small, but significant improvement in discrimination was observed when uACR, hemoglobin A1c, and social deprivation index were added. A highly significant improvement in calibration was observed when uACR was added to the base model among those with marked albuminuria. In Figure 4, we show the estimated 10-year risk of total CVD, atherosclerotic CVD, and heart failure stratified by age and sex.

**Figure 4:**
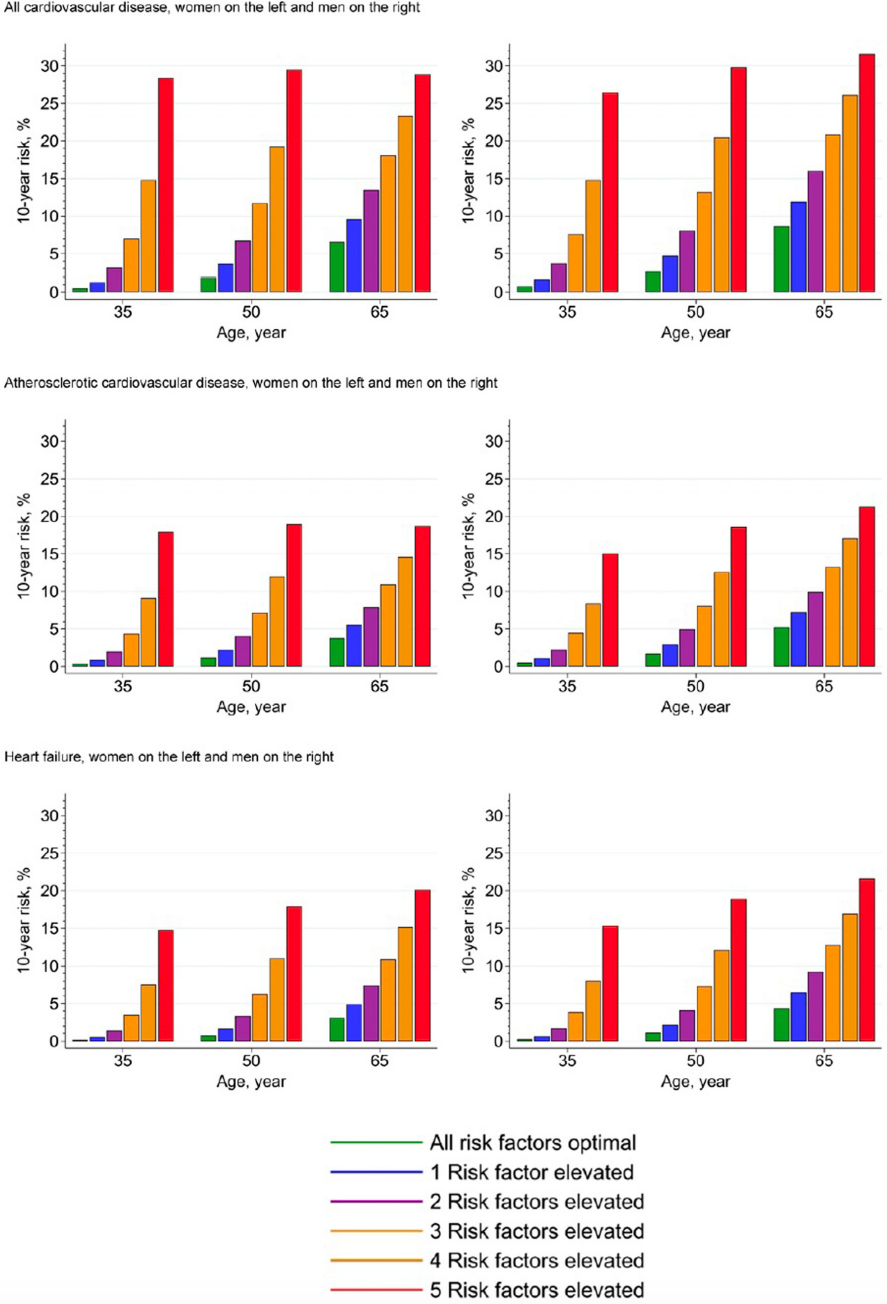
Estimated 10-year risk of total cardiovascular disease, atherosclerotic cardiovascular disease, and heart failure stratified by sex (women on the left and men on the right for each outcome) at varying ages (35, 50, and 65 years) according to the number of elevated risk factors (0–5) adjusted for competing risks of noncardiovascular disease death. Optimal risk factor levels are defined as nonhigh-density lipoprotein cholesterol (3.5 mmol/l; 135 mg/dl), high-density lipoprotein cholesterol (1.5 mmol/l, 58 mg/dl), systolic blood pressure 120 mm Hg, no diabetes, no smoking, no hypertension medications, no statin use, and estimated glomerular filtration rate 90 ml/min per 1.73 m^2^. Elevated risk factor levels included nonhigh-density lipoprotein cholesterol (5.5 mmol/l; 213 mg/dl), systolic blood pressure 150 mm Hg, diabetes, or smoking and estimated glomerular filtration rate 45 ml/min per 1.73 m^2^. For multiple elevated risk factors, the risk shown is the average risk of all combinations.

### Potential Limitations of the PREVENT Equations

The development of the PREVENT equations is a major step forward. Nevertheless, Khan *et al.*^31^ rightly noted several limitations of the risk prediction models, despite good to excellent discrimination and calibration across subgroups, including by race and ethnicity.

First, the authors used electronic medical records-based data sets, which may have excluded research-based measurements of predictors and missed adjudication of outcomes and potential for nonrandomly distributed data.

Second, they excluded people with extreme clinical values of systolic blood pressure, serum total and HDL cholesterol, or BMI. However, they pointed out that secondary analyses showed consistent risk associations between risk factors and CVD across research cohorts and electronic medical records data sets.

Third, the long baseline time period of the included data sets, spanning more than 3 decades, might have led to differences in risk factor prevalence and treatment modalities. However, secondary analyses allowed them to demonstrate the absence of differences in directionality and magnitude of hazard ratios between identified predictors and outcomes from one decade to the other.

Fourth, the authors used age for model development as the time scale. This might have led to overestimating the 30-year risk. Against this potential critique, they argued that they modeled the risk of CVD using risk factor levels at baseline and adjusted for competing risk of non-CVD death to address any potential risk overestimation.

Fifth, individual-level social determinants of health were not routinely available in all data sets. Complete consideration of such determinants among different racial and ethnic groups may lead to improved risk prediction.

Sixth, a variety of well-known biomarkers of target-organ damage were not included in the PREVENT model development. As examples, troponin and brain natriuretic peptide as markers of cardiac disease, C-reactive protein as a marker of inflammation, and coronary artery calcification as a marker of coronary artery disease were excluded. The reasons for this were their absence in present screening recommendations for primary prevention, and limited data availability in clinical data sets of large sample size.

Seventh, separate modeling was used for total CVD and its components in the development of PREVENT equations. Considering that an individual may develop 1 or more of these outcomes, the predicted risk for each composite outcome is less than the sum of its components. It may be worthwhile in future research to incorporate additional risk factors.

In addition to the limitations noted by Khan *et al.*^31^ the PREVENT equations have other potential limitations which might benefit from extended, updated versions in the future. Although the present equations cover a large age group, strictly speaking, they are valid only for individuals aged 35 to 79 years in the US. They need to be validated for younger people and ethnic groups living outside North America. Very recently, an additional study assessed the prevalence and temporal evolution of CKM syndrome stages, but it was from the US. Using the National Health and Nutrition Examination Survey (2011–March 2020), Aggarwal *et al.*^32^ found that almost 90% of US adults met the criteria for CKM syndrome (stage 1 or higher), and 15% met criteria for advanced stages, neither of which improved between 2011 and 2020. They tentatively ascribed the lack of progress, in part, to concomitant improvement and worsening of different risk factors over time.

The use of BMI as the sole obesity measure is as another potential limitation. By definition, BMI does not consider the importance of obesity distribution. Several reports have identified visceral fat as an important player in disease progression. The visceral fat index has become a potentially useful parameter in evaluating the degree of visceral, hormonally active adiposity. The calculation of visceral fat index is based on BMI, waist circumference, triglyceride, and high-density lipoprotein cholesterol. It allows identifying high-risk populations with different types of obesity. Specifically, it characterizes individuals with normal body weight, but who, like people with obesity, present with a cluster of cardiovascular risk factors including insulin resistance, impaired glucose tolerance, atherogenic lipid profiles, and hypertension.^33^ A recent study showed that high visceral fat index values were independently associated with an increased risk of incident heart failure, abnormal left-ventricular geometry, and diastolic dysfunction.^34^

Several other variables might further be considered in future adaptations, some of them reflecting more specifically either CVD, metabolic syndrome, or CKD, and others reflecting 2 or all 3 of them together. These variables include diastolic blood pressure, volume overload and edema, anemia, renin-angiotensin-aldosterone system stimulation, sympathetic nerve overactivity, CKD-mineral and bone disorder, vascular stiffness, markers of inflammation and oxidative stress, endocrine disturbances, uremic toxins, malnutrition, and sedentary lifestyle.^15^

From a more general point of view, in the future it may be more appropriate to use the term "CKM disorder" instead of the term "CKM syndrome" to describe the complex interrelationship between cardiovascular, metabolic, and kidney derangements, in analogy to the suggestion made for the proposed change of the term "cardiorenal syndrome" mentioned above.^3^

### Importance of CKM From the Nephrology Perspective

The diagnosis of CKD is often made relatively late in the course of the disease. The new CKM staging system, if broadly applied, should lead to earlier detection of CKD in individuals at risk for hypertension, CVD, metabolic syndrome, and diabetes because it recommends assessing both eGFR and uACR as early as at stage 1. By definition, individuals at that stage are characterized by the presence of obesity or dysfunctional adiposity, without evidence for CKD. Earlier detection of CKD should enable earlier, more efficacious slowing of disease progression, and consequently result in better patient outcomes, improved patient well-being, and lower health care expenditures. It could also lead to an earlier treatment of patients with CKM, using new treatments that target cardiac, kidney, and metabolic disorders such as sodium glucose transporter 2 inhibitors, glucagon-like peptide-1 agonists, and finerenone.

## Conclusion

The development of CKM syndrome with its integrated system of health stages is a useful extension from previously defined syndromes of the interrelation between cardiovascular, metabolic, and kidney diseases. The subsequently developed PREVENT models may undergo a broad application for primary prevention worldwide in people with CKM syndrome. They are based on careful calibration across racial, ethnic, and higher-risk subgroups such as CKD, obesity, and diabetes. Despite the limitations outlined above, the development of PREVENT equations is a major step forward, aimed at improving medical care for a large proportion of the general population. It remains to be seen to which extent the 10-year and 30-year risk estimates of PREVENT will translate into earlier, more appropriate treatment and prevention of CKM factors in clinical practice. Additional studies aimed at estimating short-term to medium-term risks may be more effective.

## Disclosure

ZAM reports having received grants for CKD REIN and other research projects from 10.13039/100002429Amgen, 10.13039/100004702Baxter, 10.13039/100015699Fresenius Medical Care, 10.13039/100004330GlaxoSmithKline, Merck Sharp and Dohme-Chibret, Sanofi-Genzyme, Lilly, Otsuka, Astra Zeneca, Vifor, and the French government; as well as fees and grants to charities from Astra Zeneca, Boehringer Ingelheim, and GlaxoSmithKline. TBD reports having received advisor, consultant, speaker, and/or travel fees from Astellas and Glaxo-Smith-Kline.
