# Corrigendum to “Combination of Cardiovascular, Kidney, and Metabolic Diseases in a Syndrome Named Cardiovascular-Kidney-Metabolic, With New Risk Prediction Equations” [*Kidney International Reports* Volume 9, Issue 9, September 2024, Pages 2608-2618]

**DOI:** 10.1016/j.ekir.2025.01.002

**Published:** 2025-01-03

**Authors:** Ziad A. Massy, Tilman B. Drueke

**Affiliations:** 1 Inserm Unit 1018, Team 5, CESP, Hôpital Paul Brousse, Paris-Sud University (UPS) and Versailles Saint-Quentin-en-Yvelines University (Paris-Ile-de-France-Ouest University, UVSQ), Villejuif, France; 2 Association pour l’Utilisation du Rein Artificiel dans la région parisienne (AURA), Paris, France; 3 Department of Nephrology, Ambroise Paré University Hospital, APHP, Boulogne-Billancourt, Paris, France

The authors regret that because of an issue with permissions for Figure 1, this figure and legend have now been removed from the online article, with the remaining figures renumbered with credit lines added to acknowledge permission from original sources.

The sentence containing the in-text citation to Figure 1, “In Figure 1, we show a conceptual diagram for CKM syndrome with its pathophysiological consequences reflecting multidirectional relationships among metabolic risk factors, CKD, and the cardiovascular system,” has also been replaced with, “The pathological consequences of the CKM syndrome reflect multidirectional relationships among metabolic risk factors, CKD, and the cardiovascular system.”

The authors and publisher apologize for any inconvenience caused.

DOI of original article: https://doi.org/10.1016/j.ekir.2024.05.033

